# Thermostable in vitro transcription-translation compatible with microfluidic droplets

**DOI:** 10.1186/s12934-024-02440-y

**Published:** 2024-06-10

**Authors:** Ana L. J. L. Ribeiro, Patricia Pérez-Arnaiz, Mercedes Sánchez-Costa, Lara Pérez, Marcos Almendros, Liisa van Vliet, Fabrice Gielen, Jesmine Lim, Simon Charnock, Florian Hollfelder, J. Eduardo González-Pastor, José Berenguer, Aurelio Hidalgo

**Affiliations:** 1 Centro de Biología Molecular “Severo Ochoa” (UAM-CSIC), Nicolás Cabrera 1, 28049 Madrid, Spain; 2 Instituto de Biología Molecular, Universidad Autónoma de Madrid, Nicolás Cabrera 1, 28049 Madrid, Spain; 3 Departamento de Biología Molecular, Universidad Autónoma de Madrid, Campus de Cantoblanco, 28049 Madrid, Spain; 4 Departament of Biochemistry, Cambridge University, 80 Tennis Court Road, Cambridge, CB2 1GA UK; 5 DropTech Ltd, 91 Canterbury Court, Cambridge, CB4 3QU UK; 6 Living Systems Institute, Faculty of Health and Life Sciences, University of Exeter, Stocker Road, Exeter, EX4 4QD UK; 7 Department of Physics and Astronomy, Faculty of Environment, Science and Economy, University of Exeter, Stocker Road, Exeter, EX4 4QL UK; 8 Prozomix Ltd, Building 4, West End Ind. Estate, Haltwhistle, Northumberland, NE49 9HA UK; 9 Centro de Astrobiología (CAB), CSIC-INTA, Ctra de Torrejón a Ajalvir, Km 4, 28850 Torrejón de Ardoz, Spain

**Keywords:** In vitro transcription and translation, *Thermus thermophiles*, Droplet microfluidics, Cell-free protein expression, Thermozymes

## Abstract

**Background:**

In vitro expression involves the utilization of the cellular transcription and translation machinery in an acellular context to produce one or more proteins of interest and has found widespread application in synthetic biology and in pharmaceutical biomanufacturing. Most in vitro expression systems available are active at moderate temperatures, but to screen large libraries of natural or artificial genetic diversity for highly thermostable enzymes or enzyme variants, it is instrumental to enable protein synthesis at high temperatures.

**Objectives:**

Develop an in vitro expression system operating at high temperatures compatible with enzymatic assays and with technologies that enable ultrahigh-throughput protein expression in reduced volumes, such as microfluidic water-in-oil (w/o) droplets.

**Results:**

We produced cell-free extracts from *Thermus thermophilus* for in vitro translation including thermostable enzymatic cascades for energy regeneration and a moderately thermostable RNA polymerase for transcription, which ultimately limited the temperature of protein synthesis. The yield was comparable or superior to other thermostable in vitro expression systems, while the preparation procedure is much simpler and can be suited to different *Thermus thermophilus* strains. Furthermore, these extracts have enabled in vitro expression in microfluidic droplets at high temperatures for the first time.

**Conclusions:**

Cell-free extracts from *Thermus thermophilus* represent a simpler alternative to heavily optimized or pure component thermostable in vitro expression systems. Moreover, due to their compatibility with droplet microfluidics and enzyme assays at high temperatures, the reported system represents a convenient gateway for enzyme screening at higher temperatures with ultrahigh-throughput.

**Supplementary Information:**

The online version contains supplementary material available at 10.1186/s12934-024-02440-y.

## Background

Cell-free protein synthesis (CFPS) involves the utilization of the transcription and translation machinery of the cell to produce proteins of interest, independently of constraints imposed by cellular viability and variability, as well as membrane integrity [1]. This acellular paradigm allows a higher tolerance for toxic substrates or products, easy manipulation and a more precise control over both the synthetic components and the synthesized products, especially in complex biological networks, such as gene circuits or metabolic pathways [2–4]. In fact, cell-free systems also allow better control over the experimental parameters and the customization of one or more steps in the workflow of protein synthesis, such as transcription, translation or post-translational modifications even at high-throughput.

Whereas in vivo protein synthesis is coupled to the cellular ATP/GTP pools, in a cell-free context energy-rich molecules must be provided by very expensive, high-energy phosphate compounds and auxiliary enzymes, such as those involved in glycolysis and the Krebs cycle. In spite of this limitation, in vitro protein synthesis has rapidly found its way from basic research into application, mostly owing to its reproducibility and controlled environment. Noteworthy uses of CFPS in basic research include the synthesis of protein libraries for functional genomics and structural biology and the production of functional membrane proteins, but one of the most outstanding applications is the production of biopharmaceuticals such as antimicrobial peptides, cytokines, vaccines and antibodies [5].

Several cell-free expression systems have been developed (reviewed in [6]), e.g. from *Escherichia coli*, wheat germ, yeast, rabbit reticulocyte, HeLa cells, insect *Spodoptera frugiperda* 21 (*Sf*21), Chinese hamster ovary cells, tobacco BY-2 and *Leishmania tarentolae.* Further bacterial examples include extracts derived from *Streptomyces venezuelae* [7], *Bacillus subtilis* [8] and *Vibrio natriegens* [9]. Each of the above mentioned CFPS systems has different advantages and limitations and therefore, the choice of source depends on the nature of the protein to be expressed and of downstream applications. In fact, an attempt at wide-range, cell-free expression systems has been developed that combines cell lysates from 10 diverse bacterial species [10].

While the vast majority of the CFPS systems reported are based on cellular extracts, cell-free expression systems based on a mixture of tRNAs, ribosomes and recombinantly expressed, pure, histidine (His)-tagged components for transcription, translation and energy generation have been reported for *E. coli* [11] and *Thermus thermophilus* [12]. The reconstituted *T. thermophilus* system is not commercially available but the highly optimized PURE (Protein synthesis Using Recombinant Elements) system from *E. coli* has been commercialized as NEB PURExpress^®^, in which in vitro transcription and translation (IVTT) are carried out in a one-step reaction. In this system, competing side reactions (e.g. nucleases, proteases, phosphatases) are missing, compared with traditional S30 cell extracts where all soluble cytosolic components are present. Thus, linear nucleic acids, such as PCR products and mRNAs, the synthesized proteins and phosphorylated energy sources are more stable in PURE systems. However, important cofactors or chaperones necessary for efficient folding of the target protein may also be missing, which together with its high cost, represent the major disadvantages of the PURE system. On the other hand, crude cell extracts represent an inexpensive route to cell-free protein synthesis, reaching recombinant protein yields of up to 2.35 mg/ml [13], which makes extract-based CFPS a better candidate than the PURE system for scalability into high-volume fermentation conditions [14].

The majority of CFPS systems available are active at moderate temperatures (20–40 °C), but the functional expression of highly thermostable proteins that do not fold properly at room temperature requires protein synthesis at higher temperatures. To this end*,* Ruggero et al. reported an efficient in vitro translation (IVT) of archaeal natural mRNAs at 75 °C, with extracts of the extreme thermophile *Sulfolobus solfataricus* [15]. Endoh and coworkers reported another IVT system, combining extracts from the hyperthermophilic archaeon *Thermococcus kodakarensis* KOD1. In vitro protein synthesis at high temperatures has also been demonstrated in vitro for thermophilic bacteria. Ohno‐Iwashita *et al*. reported IVT of poly(Phe) with a cell-free extract of *T. thermophilus* at 65 °C [16] and more recently, Zhou and coworkers described IVT of superfolder GFP (sGFP) using purified thermostable components [12].

The absence of a physical boundary represents a significant challenge in the application of IVTT to analyze individual gene variants from metagenomic or protein variant libraries for enzyme discovery and evolution, respectively. The compartmentalization of the IVTT reaction is strictly needed to establish a linkage between the desired phenotype and its encoding genotype. Furthermore, given the high cost of IVTT reactions and the throughput required to analyse at least a fraction of the genetic diversity contained in (meta) genomic libraries, even the use of the smallest microwell plates would compromise the economic viability of the screening campaign. However, compartmentalizing IVTT systems within monodisperse aqueous droplets with cell-like diameters (20–200 μm) and femto- to nanoliter volumes dispersed in an immiscible perfluorinated hydrocarbons, may unlock the economic feasibility of in vitro screenings. In fact, large numbers of droplets (~ 10^7^ −10^9^ in one experiment) can be produced at a more reduced cost per assay (~ 10^6^ -fold) [17] than industrial robotic screening platforms. Among the successful experiments in directed evolution that involve in vitro expression in microdroplets are selections of enzymes such as DNA methyltransferases, phosphotriesterases, or glycosidases [18–20] in polydisperse droplets and more recently, proteases using monodisperse droplets created with microfluidics [21]. Since the success of such directed evolution experiments in droplets is dependent on the efficiency of IVTT, protein expression can be boosted by generating multiple copies of the DNA template via amplification of encapsulated single DNA molecules on beads [22] or by rolling circle amplification (RCA) prior to IVTT and a fluorometric enzyme assay [21].

Considering thermostability as an essential property of enzymes in industrial processes, this work aimed to develop thermostable cell-free protein expression system for thermozymes that: i) enables a one-pot protein synthesis coupled to an enzymatic assay and ii) is compatible with droplet microfluidics. To the best of our knowledge, this is the first description of enzyme assays powered by an IVTT reaction at high temperature, which is also compatible with microfluidic droplets. These findings will undoubtedly provide a cost-effective, simple and powerful tool for ultrahigh-throughput screening of libraries for enzyme discovery and evolution, as well as opportunities in different applications from protein biochemistry to biomedical science or extremophile research.

## Methods

### Strains and growth media

*E. coli* was grown at 37 °C in Luria–Bertani lysogeny broth (LB; 10 g/L Tryptone, 10 g/L NaCl and 5 g/L yeast extract) and *T. thermophilus* was grown at 65 °C in *Thermus* Broth (TB; 8 g/L Tryptone, 4 g/L NaCl and 3 g/L yeast extract in carbonate-rich mineral water). Media were solidified by the addition of 2% (w/v) of agar, if needed, and/or supplemented with a final concentration of 30 µg/ml kanamycin (Kan), 100 µg/ml ampicillin (Amp) or 20 µg/ml chloramphenicol (Cam) for selection.

*E. coli* DH5*α* [*sup*E44, Δ*lac*U169 (φ80 *lacZ*ΔM15), *hsd*R17, *recA*, *end*A1, *gyr*A96, *thi*^−1^ *relA1*] was used for construction of plasmids, whereas electrocompetent *E. coli* BL21(DE3) [*hsd*S, *gal* (λcIts857, *ind*1, *Sam7*, *min5*, *lacUV5*-T7 gene 1] and *E. coli* BL21 Rosetta [F^−^ *ompT hsdS*_*B*_ (r_B_^−^m_B_^−^) *gal dcm* (DE3) pRARE (Cam^R^)] were used for overexpression and purification of recombinant proteins.

*T. thermophilus* strain HB27 was used to develop and optimize the IVTT protocol and the composition of CFPS reaction mixes. To reduce the background activity when coupling IVTT with enzymatic activity, S30 extracts were generated with *T. thermophilus* strain BL03, deficient in major hydrolytic activities including the gene encoding glycosidase *TTP0042* [23].

### Nucleic acid manipulation and transformation

Isolation of plasmid DNA was carried out with Genejet Plasmid Miniprep kit (Thermo Fisher Scientific, Pittsburgh, PA, USA), according to the manufacturer´s instructions. DNA was amplified by polymerase chain reaction (PCR), using GoTaq Flexi Polymerase (Promega, Madison, WI, USA), while for high fidelity amplification, PfuUltra II HS polymerase (Agilent Genomics, Santa Clara, CA, USA) was employed, following the manufacturer's protocol. DNA fragments such as PCR products or DNA fragment as an agarose gel slices were purified using the Wizard® SV Gel and PCR Clean-Up System kit from Promega (Madison, WI, USA). DNA was digested with the appropriate restriction endonucleases (FastDigest, Thermo Fisher Scientific, Pittsburgh, PA, USA) following the manufacturer's instructions. To avoid religation, vector DNA was dephosphorylated using FastAP Thermosensitive Alkaline Phosphatase (ThermoFisher Scientific, Pittsburgh, PA, USA). For routine plasmid construction, digested vectors and insert DNA fragments were ligated using T4 DNA Ligase (Promega, Madison, WI, USA) following manufacturer's recommendations. DNA concentration was measured with Nanodrop™ One (Thermo Fisher Scientific, Pittsburgh, PA, USA). Sanger DNA sequencing was performed by Macrogen Inc. (Seoul, Republic of Korea) and DNA sequences were analyzed using SnapGene software (GSL Biotech, Boston, MA, USA).

The genes encoding pyruvate kinase (PK, gene name *TT_C1611,* UniProt ID: Q72H84), nucleoside diphosphate kinase (NDK, *TT_C1798,* Q72GQ0), adenylate kinase (ADK, *TT_C1307,* Q72125), lactate dehydrogenase (LDH, *TT_C0748,* P62055) and inorganic pyrophosphatase (IPP, *TT_C1600,* Q72H95) were amplified by PCR from *T. thermophilus* HB27 genomic DNA extracted using the DNeasy UltraClean MicrobialKit (QIAGEN). Amplified genes were digested and inserted into the pET28b( +) vector (Merck Millipore) (NDK, ADK, PK, IPP) or pET28b( +) vector (Merck Millipore) (LDH). LDH, PK, NDK and IPP were cloned with NdeI and HindIII restriction enzymes (ThermoFisher Scientific, Pittsburgh, PA, USA), whereas ADK was cloned with NdeI and EcoRI (ThermoFisher Scientific, Pittsburgh, PA, USA). Chemically competent *E. coli* DH5α cells were transformed with the constructs. Transformation was carried out by heat shock following the method described by Hanahan [24]. Five microliters of ligation product or 100–200 ng of plasmid was added to a 50 µl of competent cells, which were further incubated on ice for 30 min. Then, the tubes were heated at 42 °C for 90 s. After cooling down on ice for 5 min, 350 µl of SOC medium was added and incubated in a shaker at 37 °C for 1 h for the expression of ampicillin or 3 h for the expression of kanamycin resistance.

Clones were checked by restriction digestion with the corresponding enzymes, followed by sequencing. To ensure correct in-frame expression of the His_6_-tag-encoding sequence, ADK required additional site-directed mutagenesis, performed using QuikChange II XL Site-Directed Mutagenesis Kit (Agilent Technologies, Santa Clara, CA, USA) followed by transformation of *E. coli* DH5α cells and confirmative sequencing.

The gene encoding superfolder GFP (sGFP) was amplified by PCR using plasmids pET28b ( +)_sGFP as template (Supplementary Table 1) and the T7 primers indicated in Supplementary Table 2.

### Solutions and chemicals

All IVTT buffers and solutions were prepared with diethylpyrocarbonate (DEPC)-treated water (0,05%) or nuclease-free water (Invitrogen, CA, USA). The S30A and S30B buffers along with the HEPES, nucleotide mix, potassium glutamate, magnesium glutamate, folinic acid and spermine solutions were prepared following the protocol described in Sun et al., 2013 [25]. The amino acid solution was prepared according to Cashera & Noireaux, 2015 [26].

Chemicals were purchased in analytical grade from Merck KGaA (Darmstadt, Germany), Bio-Rad Laboratories (Hercules, CA, USA), or Sigma-Aldrich (St. Louis, MO, USA). Sigma-Aldrich synthesized the oligonucleotides used.

### Protein expression and purification

Electrocompetent *E. coli* BL21 (for ADK, PK, NDK and IPP) and *E. coli* BL21 Rosetta (for LDH) cells were transformed with the previously described constructs. Electroporation was carried out by mixing 45 µl of competent cells with 100–200 ng of plasmid and subjecting the cells to a short 5 ms electric pulse under a 12500 V/cm electric field in a Gene Pulser II^®^ (Bio-Rad, Hercules, CA, USA) (2500 v, 201 Ω and 25 µF) using 0.2 cm gap cuvettes (Bio-Rad Gene Pulser). Immediately after the pulse, 500 µl of SOC medium as added and incubated at 37 °C during the appropriate time for each antibiotic resistance before plating on selective medium.

Transformant colonies were grown overnight in 20 mL LB medium supplemented with ampicillin (pET22b constructs) or kanamycin (pET28b constructs). Chloramphenicol was supplemented when using *E. coli* BL21 Rosetta as a host. Cultures were inoculated at a 1/100 dilution of those preinocula and induced at OD_600_ 0.5 with 0.5 mM IPTG for PK, IPP, NDK and ADK. LDH-transformants were induced at OD_600_ 0.7 and 1 mM of IPTG. Cells were grown at 37 ºC for 5 h after induction (PK, PPI, NDK) or 20 h at 22 ºC (ADK). *E. coli* BL21 Rosetta transformed with LDH was grown for 36 h at 17 ºC. Cells were harvested and pelleted after induction and growth. Pellets were frozen and stored at −20 ºC until further use. Negative controls were carried out with transformants harboring the corresponding empty vectors.

To verify the expression of proteins, pellets from different times after induction were resuspended in 50 mM phosphate buffer, sonicated (0.6 Amplitude and 50% pulse) and centrifuged at 14,100 × g for 10 min. The supernatant was separated and the pellet was washed with phosphate buffer 50 mM pH 7.5 and 0.1% w/v Triton X-100, analyzed by SDS-PAGE in a 12% acrylamide gel according to the method described by Laemmli [27]. The gels were stained with Coomassie Brilliant Blue G-250 from Bio-Rad Laboratories (Hercules, USA).

To purify ADK, NDK, PK and IPP, pellets from large scale production were resuspended in 50 mM phosphate buffer, sonicated (0.6 Amplitude and 50% frequency) and centrifuged at 15000 × g for 30 min. Proteins found in the soluble fraction were purified by immobilized metal ion affinity chromatography (IMAC), using Talon resin (BD ClonTech). Purified proteins were concentrated using 10 kDa cutoff Amicon Ultra Centrifugal Filters (Merck Millipore) and stored in 50% v/v glycerol at -20 ºC until further use. After protein concentration, purity was checked by sodium dodecyl sulfate polyacrylamide gel electrophoresis (SDS-PAGE) in a 12% polyacrylamide gel using the Bio-Rad Protein Assay (Bio-Rad, Hercules, CA, USA), according to the manufacturer’s protocol, using bovine serum albumin (BSA) as standard.

### Western blot

Western blot analysis was carried out by semidry electroblotting on immobilon-P transfer membrane from Merck KGaA (Darmstadt, Germany). Anti-GFP polyclonal antibody and secondary goat-anti-rabbit HRP conjugate antibody were supplied by Thermo Scientific (Ulm, Germany) and used at the dilutions recommended by the manufacturer. The antibodies were diluted according to manufacturer´s recommendations. Detection of proteins was achieved by chemiluminescent reaction and capture on a film, using the charge-coupled device “X-OMAT 2000 Processor” from Kodak (Rochester, USA). The reaction was enabled by addition of a solution containing 100 mM Tris–HCl pH = 7.8, luciferin and luminol and a second solution containing 100 mM Tris–HCl pH 7.8 and H_2_O_2_ (enhanced chemiluminescent reaction). Quantification of sGFP was carried out by image analysis using Fiji image processing software [28] by comparison of lanes containing 8 μl of completed IVTT reaction with lanes containing 300 or 400 ng of pure sGFP.

### Activity assays of the energy regeneration enzymes

Enzymatic assays were performed at 60 ºC. Four replicates of each sample in the endpoint assays and three replicates in the real-time assays were measured to assure statistical significance. Negative controls were performed by omitting the substrate. Spontaneous conversion without the enzyme was also performed as a negative control.

LDH was assayed at 60 ºC in 50 mM Tris–HCl buffer, 0.2 mM NADH and 0.3 mM pyruvate. The assay was performed at pH values ranging from 5.5 to 8 and at different ionic strength values ranging from 4 to 40 mM [29]. One unit of LDH activity was defined as the amount of enzyme able to convert 1 micromole of pyruvate per minute.

PK was assayed with 5 mM phosphoenolpyruvate (PEP), 5 mM ADP, 20 mM KCl and 5.4 mM MgSO_4_ in 100 mM Tris buffer pH 7.5. In this case, PK was assayed using a coupled assay with LDH and recording NADH absorbance at 340 nm with 5 mM ADP, 40 mM KCl, 40 mM MgSO_4_, 6 mM PEP, 0.4 mM NADH and 6.3 mU of LDH in 50 mM Tris buffer pH 7 [30]. One unit of PK activity was defined as the amount of enzyme able to convert 1 micromole of PEP per minute.

NDK was assayed with 40 mM KCl, 40 mM MgSO4, 6.3 mU LDH, 7.06 mU PK, 1 mM GDP, 0.2 mM NADH, 1.1 mM PEP and 2.2 mM ATP in 50 mM Tris–HCl buffer pH 7. One unit of DNK activity was defined as the amount of enzyme able to convert 1 micromole of GDP per minute.

ADK was assayed with 2.5 mM ADP, 100 mM KCl and 2 mM MgCl_2_ in 50 mM Tris–HCl buffer pH 7.5. Different dilutions of the enzyme preparation were assayed for 1 h and analyzed with a luciferin-luciferase coupled reaction [31] using the CLSII-Bioluminiscent ATP Assay Kit (Roche).

IPP was assayed with 5 mM pyrophosphate (PPi), 120 mM NaCl, 5 mM KCl and 5 mM MgCl_2_ in 20 mM Tris–HCl buffer pH 7.5. For the end-point assay different dilutions of the enzyme were assayed and stopped at different times [32]. The released phosphate was analyzed with the malachite-green phosphomolybdate assay by adding 200 μl of the malachite green reagent (5.72% w/v ammonium molybdate in HCl 6 M, 2.32% w/v PVA, 0.0812% w/v malachite green; 1:1:2:2) and measured at 640 nm [33].

### In vitro transcription and translation extracts

Preparation of S30 extracts was performed by a modification of the method published by Sun et al*.* [25] under RNase-free conditions. Briefly, a 30 ml preculture of *T. thermophilus* HB27 was grown overnight aerobically under rotational shaking (150 rpm) at 65 °C in liquid TB [34]. The preculture was used to inoculate 1.2 L culture with TB medium which was cultured under aerobic conditions at 65 ºC until A600 reached 1.0–1.2 (corresponding to the mid-log growth phase). Immediately after growth the culture was cooled down to 4 ºC. Cells were harvested by centrifugation at 5000 × g for 15 min at 4 ºC and washed with 500 mL of DEPC-treated water (0.05%). The cell pellet was resuspended in 250 mL of buffer S30A (14 mM Mg-glutamate, 60 mM K-glutamate, 50 mM Tris, pH 7.7, 2 mM DTT) and centrifuged once more at 5000 × g for 15 min. Fifty milliliters of S30A buffer was used to wash the cells one last time. The pellet was weighed before being stored at −80 °C. The cell pellet was transferred into a prechilled mortar and disrupted by grinding with 1.5 g of precooled alumina (Sigma‒Aldrich, St. Louis, MO, USA) per gram wet weight for 15 min over ice. The cell slurry was resuspended in buffer S30A (0.5 mL per gram wet weight) and transferred to a centrifuge tube. The extract was centrifuged at 30,000 × g for 30 min at 4 °C to remove alumina and cell debris. The resulting supernatant was incubated at 37 °C for 80 min with shaking at 200 rpm and then centrifuged at 12000 × g for 10 min at 4 °C. The S30 extract was dialyzed against S30B buffer (14 mM Mg-glutamate, 60 mM K-glutamate, ~ 5 mM Tris, pH 8.2, 1 mM DTT) in 3 k MWCO dialysis cassettes (Thermo Fisher Scientific, Pittsburgh, PA, USA) for 3 h at 4 ºC. The extract concentration was determined by Bradford (Bio-Rad, Hercules, CA, USA). Aliquots of the S30 cell extract were then frozen in dry ice and stored at −80 °C.

### In vitro transcription and translation in bulk

The CFPS reaction was performed in a final volume of 25 µl using plasmid DNA as a template, unless stated otherwise. The reaction mixture contained the S30 extracts and the IVTT buffer mix with the ingredients shown in Table 1 and a thermostable T7 RNA polymerase (tT7 RNApol) (Toyobo). RNase inhibitor was purchased from New England Biolabs (Ipswich, MA). ATP, GTP, UTP and CTP were purchased from Promega (Madison, WI). Total tRNAs from *E. coli* were purchased from Sigma-Aldrich (St. Louis, MO).

**Table 1:** Composition of the reaction mix for thermostable IVTT

		Final concentration
Buffer Mix	HEPES–KOH (pH = 8)	50 mM
	Magnesium glutamate (MgGlut)	10 mM
	Potassium glutamate (KGlut)	100 mM
	Dithiothreitol (DTT)	1.5 mM
	Amino acids (aa mix)	1.5 mM
	Phosphoenolpyruvate (PEP)	15 mM
	Pyruvate kinase (PK)	0.22 μM
	Nucleoside diphosphate kinase (NDK)	0.070 μM
	Inorganic pyrophosphatase (PPA)	0.040 μM
	Adenylate kinase (ADK)	0.26 μM
	Folinic acid	0.068 mM
	Murine RNase inhibitor	1 U/μl
	Total *E. coli* tRNAs	0.2 mg/ml
	ATP/GTP	2 mM
	UTP/CTP	1.2 mM
	Spermine	2 mM
Template DNA		40 ng/μl
Thermostable T7 RNA polymerase		1 U/μl
*T. thermophilus* S30 extract		4.7 mg/ml

When the IVTT was coupled to an enzymatic activity assay, a fluorogenic substrate was added to the medium at a final concentration of 5 µM from a 10 mM stock prepared by dissolving substrates in DMSO. The substrates used were fluorescein di-β-D-glucopyranoside (FDGlu), fluorescein di-β-D-cellobioside (FDC) purchased from Thermo Fisher Scientific and AAT Bioquest, respectively. The samples were transferred to a real-time PCR cycler (Rotorgene 6000, Corbett Research) and incubated at the indicated temperatures for the indicated time. Fluorescence was measured in the high-resolution melt (HRM) channel (excitation 460 ± 20 nm, emission 510 ± 5 nm) and the gain was adjusted manually according to the fluorescence reading during the first ten cycles of the reaction.

### In vitro transcription and translation at different temperatures

Cell-free transcription and translation reactions were carried out using PURExpress^®^ In Vitro Protein Synthesis Kit (NEB) following the manufacturer´s protocol, adjusted to 10-µl reaction volume and using 5 ng of pET28_*sGFP* as a template. Fluorescence was measured using a Rotorgene 6000 real-time thermocycler (Corbett Research) in the high-resolution melt (HRM) channel (excitation 460 ± 20 nm, emission 510 ± 5 nm) and the gain was adjusted manually according to the fluorescence reading during the first ten cycles of the reaction. The reaction was performed at 37 ºC, 40 ºC, 45 ºC and 50 ºC.

### Rolling circle amplification (RCA) coupled with IVTT

Plasmid DNA was amplified using REPLI-g^®^ Midi Kit (QIAGEN), according to the manufacturer´s instructions. One nanogram of template plasmid was used in 25 µl final volume and the reactions were incubated isothermally at 30 °C for 3 h, followed by inactivation at 65 °C for 3 min. Then, components for IVTT were added to the reaction, either *T. thermophilus* extracts or PURExpress^®^ In Vitro Protein Synthesis Kit (New England Biolabs, Ipswich, MA, USA) and the reactions were further incubated at either 50 °C or 37 °C for another 4 h in the case of *T. thermophilus* extracts or 2 h for the *E. coli* PURE system.

### In vitro transcription and translation in droplets

Designs of flow focusing chips used for droplet generation were obtained from DropBase (https://openwetware.org/wiki/DropBase:Devices). Device master molds were microfabricated by Tekniker (Eibar, Spain) on silicon wafers by soft photolithography with a 30 µm height. A mixture of poly(dimethylsiloxane) (PDMS, Sylgard 184 Dow Corning (Midland, USA) and cross linker (ratio 10:1 w/w) was poured over the master mold, then degassed and cured overnight at 65 ºC. The cured device was cut and peeled from the master, and holes for tubing were cut with a 1-mm biopsy punch (Kai Medical, Solingen, Germany). After treatment with oxygen plasma for 15 s (Diener Femto, 30 W, 40 kHz), the device was sealed against a glass slide. The channels were washed with a 1% (v/v) solution of trichloro(1H,1H,2H,2H-perfluorooctyl)silane in HFE 7500 and baked for at least 3 h at 65 ºC.

The flow was driven with Nemesys Base 120 module and Nemesys S pumps (Cetoni GmbH (Korbußen, Germany) syringe infusion pumps using 1 mL and 100 μL gastight syringes, (Hamilton, Reno, USA) connected to fine-bore polyethylene tubing with 1.09 mm outer diameter and 0.5 mm inner diameter (Smiths Medical). We tested several combinations of surfactants Pico-Surf (Sphere Fluidics) or RAN-008 Fluorosurfactant (RAN Biotechnologies) with fluorocarbon oils HFE7500, FC70 or FC40 (Merck Millipore) as the continuous phase. The microfluidic equipment was integrated by an inverted microscope (Leica DMi8) connected to a high-speed camera (Fastcam Mini UX 50, Photron) for real-time visualization of experiments. Emulsions were routinely photographed in Fast-Read 102 slides with counting chambers (Biosigma s.r.l., Italy) using an Olympus BX50 microscope equipped with a Pike F-032B camera (Allied Vision Technologies) and a 25 × objective. Dimensions were determined from at least n = 40 droplets using Fiji image processing software [28].

## Results

### Coupled transcription and translation at high temperature

We generated S30 extracts from *T. thermophilus* cells, modifying an existing protocol for *E. coli* under RNase-free conditions [25]. We also supplemented with DTT, which helps the folding of proteins that require the formation of disulfide bonds for activity and spermine to increase the speed of peptide synthesis [35]. Additionally, tRNAs from *E. coli* (Merck) and tT7 RNApol (Toyobo) were added to the mix. Finally, we added a thermostable multienzymatic ATP-recycling cascade composed of pyruvate kinase (PK), adenylate kinase (ADK) and inorganic pyrophosphatase (IPP) and a GTP-recycling cascade composed of pyruvate kinase (PK) and nucleoside diphosphate kinase (NDK), all from *Thermus thermophilus* (Supplementary Table 3). Protein expression was initiated by the addition of a suitable template and we chose the superfolder GFP (sGFP) as the target protein since it is able to properly fold and fluoresce when expressed in *T. thermophilus* at 70 °C [36].

**Fig. 1:**
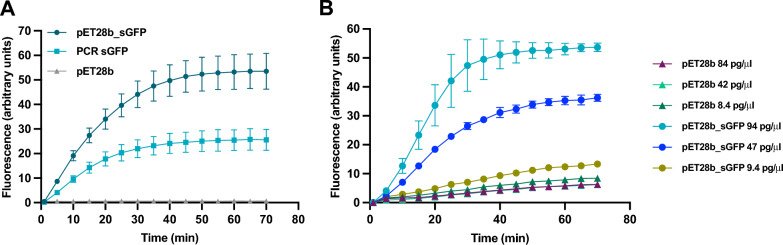
Synthesis of sGFP using *T. themophilus* S30 extracts and different types of DNA template. **A** Reaction mixtures containing 40 ng/µl of pET28b_*sGFP* (circles) or the same molar concentration of sGFP PCR amplicon (squares) were incubated at 50 °C for 70 min. As a negative control of the reaction, an empty pET28b vector was used (triangles). Composition of the reaction mixtures are indicated in Table 1. sGFP synthesized was monitored in real time as fluorescence emission. **B** Different amounts of template DNA (in ng/ml) were tested as in (**A**), with the corresponding amounts of empty vector as negative controls. Results are the average of n = 3 reactions and error bars represent standard deviations

Then, to determine the optimal type of template to use in our cell-free expression system, different types of DNA were added to the IVTT mix, the reactions were incubated at 50 °C for 120 min and sGFP expression levels were analysed and compared (Fig. 1A). We tested the 957 bp amplicon of the sGFP gene (PCR sGFP, linear DNA), a pET28b( +) plasmid harboring the sGFP-encoding gene (pET28b_sGFP, circular DNA) under the control of the strong φ10 promoter for the T7 RNA polymerase (T7 promoter) and an empty pET28b( +) vector as negative control of the reaction. Upon mixture and incubation of all components in a real-time thermocycler, a rapid accumulation of protein product was observed after 20 min of reaction and reached saturation at 40 min when circular DNA was used as template (Fig. 1A). However, we observed an approximately threefold lower amount of synthesized protein when linear DNA was used as a template. We did not detect any fluorescence when the empty vector was used as a template, as expected. From this result, we concluded that the extracts are functional and that circular DNA templates are preferred.

To determine the sensitivity of our system, we decreased the concentration of circular DNA template concentration. We tested 94, 47 and 9.4 pg/μl pET28b_sGFP DNA and the same number of molecules of empty pET28b( +) (amounting to 84, 42 and 8.4 pg/μl), as negative control. We were able to detect fluorescence in the presence of DNA amounts as low as 9.4 pg/μl using a real-time thermocycler (Fig. 1B). A minimal fluorescence signal was detected when an empty plasmid was used in the reaction, likely due to the experiment being performed at the maximum instrument gain used to detect even the lowest amount of sGFP produced.

**Fig. 2:**
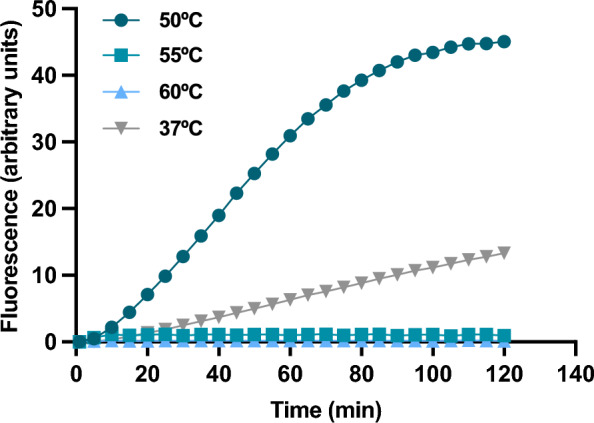
Temperature limit of sGFP synthesis with *T. thermophilus* S30 extracts. Reaction mixtures containing 40 ng/µl of pET28b_*sGFP* were incubated at 37 °C (inverted triangles), 50 °C (circles), 55 °C (squares) and 60 °C (triangles) for 120 min. Composition of the reaction mixtures are indicated in Table 1. sGFP synthesized was monitored in real time as fluorescence emission

We also explored the temperature limit of the thermostable in vitro transcription and translation system. To that end, the reaction mixture using pET28b_sGFP as template was incubated for 2 h at different temperatures ranging from 37 °C to 60 °C and particularly, above and below the optimum for the tT7 RNApol. The maximum levels of protein synthesis were observed at 50 °C and some synthesis was even detected at 37 °C (Fig. 2). However, no significant synthesis of sGFP was observed when the reactions were carried out at or above 55 °C.

Given the fact that increasing the temperature above 50 °C was not conducive to increasing the yield, we tried to increase the amount of tT7 RNA polymerase instead. When sGFP synthesis was carried out in the presence of different amounts of tT7 RNA polymerase (between 0.5 and 2.5 U/µl) and incubated at 50 °C for 2 h, we did not observe a linear increase in protein yield (Supplementary Fig. 1).

**Fig. 3:**
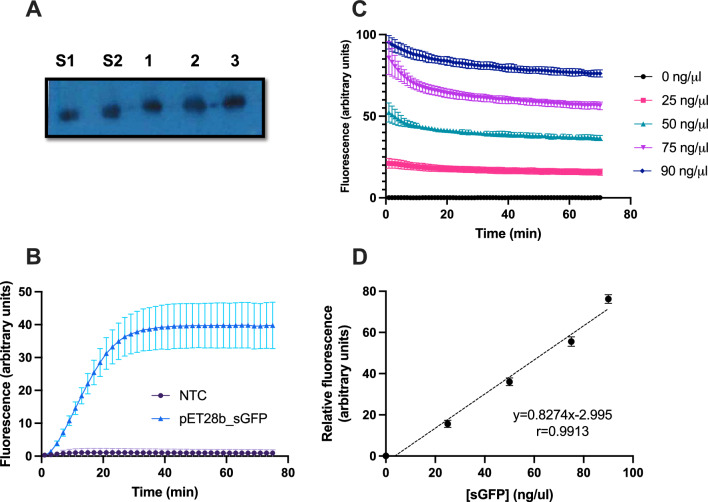
Quantification of the yield of CFPS reactions. Reaction mixtures containing 40 ng/µl of pET28b_*sGFP* as template were incubated at 50 °C for 70 min in a real-time thermocycler and the yield of sGFP was quantitated independently using both Western Blot and a calibration curve of sGFP. **A** Western blot analysis of 8 μl aliquots of three independent reactions using an anti-GFP antibody and the ECL developing reaction, S1: 300 ng purified sGFP as standard, S2: 400 ng purified sGFP, 1- 3: three replicate CFPS reactions. **B** Average progress of the 3 independent CFPS reactions analyzed by Western Blot in panel A. **C** Fluorescence of sGFP standards determined in triplicate after incubation for 70 min at 50 °C in a real-time thermocycler; **D** calibration curve of sGFP standards after 70 min incubation at 50 °C. Error bars represent standard deviation

The amount of synthesized sGFP after completion of IVTT at the optimum temperature of 50 °C was quantitated both by image analysis of a Western blot comparing with known amounts of sGFP (Fig. 3A) and by interpolating the fluorescent signal registered in a real-time thermocycler with a sGFP calibration curve acquired with the same parameters (Fig. 3B–D). The former method yielded 67.7 ng/µl sGFP whereas the latter yielded 51.8 ng/µl from the same IVTT reaction. Other IVTT reactions carried out and acquired under the same conditions yielded 68.3 ng/µl (Fig. 1) and 58.1 ng/µl (Fig. 2), which are in a similar range.

### Coupling cell-free protein synthesis with DNA amplification

To further increase sensitivity, we sought to couple DNA amplification with IVTT. Since plasmid DNA is the preferred template (Fig. 1A), we chose isothermal, random and multiple-primed rolling circle amplification (RCA) using Φ29 DNA polymerase to perform the amplification of template DNA. In our case, RCA and IVTT must be necessarily performed sequentially due to the differences in the stability of the enzymes and components responsible for DNA amplification and IVTT.

To examine whether RCA products can serve as template for the *T. thermophilus*-based CFPS system, we selected the REPLI-g® Midi Kit (QIAGEN) for DNA amplification and *T. thermophilus* extracts described in this work or commercially available *E. coli* PURExpress^®^ (NEB) for the IVTT step, using 1 ng of pET22b_sGFP as input DNA. To reduce the cross-inhibition between RCA and IVTT and, in accordance to similar assays in the literature [21], we tested different RCA:IVTT volumetric ratios (1:1, 1:2 and 1:5). To determine the contribution of the input plasmid (1 ng) to the total protein synthesis yield, a control reaction was carried out without the addition of the RCA components. When reactions were performed using the *E. coli* reconstituted system, significant levels of protein synthesis were detected after 30 min incubation, in particular when a 1:5 RCA:IVTT ratio was used (Supplementary Fig. 2A). However, no improvement in yield was obtained when IVTT was performed with *T. thermophilus* extracts at 50 °C (Supplementary Fig. 2B).

### Coupling cell-free protein synthesis with enzyme activity assays

**Fig. 4:**
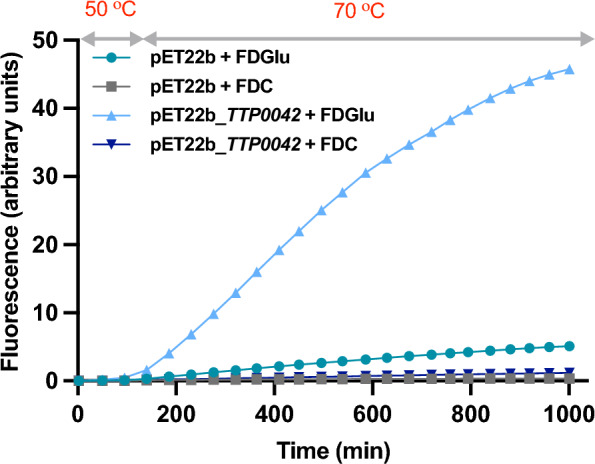
Coupling cell-free protein synthesis with an enzymatic activity. Reaction mixtures containing 40 ng/µl of pET22b_*TTP0042* and 5 µM of either FDGlu (triangles) or FDC (inverted triangles) were first incubated at 50 °C for 2 h and then, temperature was increased to 70 °C for 17 h. An empty pET22b plasmid was incubated in the presence of FDGlu (circles) or FDC (squares) as negative controls of the reactions. Composition of the reaction mixtures are indicated in Table 1

Although we have shown that *T. thermophilus*-based CFPS system allows the synthesis of proteins at high temperature (Figs. 1 and 2), library screening for enzyme discovery or evolution requires carrying out an enzyme assay either simultaneously or directly after protein synthesis, preferably in a one-pot setup. Consequently, we performed a coupled protein synthesis and activity assay, in which either fluorescein di-β-D-glucopyranoside (FDGlu) or fluorescein di-D-β-cellobioside (FDC) was added to the IVTT mix. The reaction was started by the addition of the pET22b vector harboring a gene coding for a promiscuous glycosidase (TTP0042) from *T. thermophilus* HB27 [37] as template. Since the optimum temperature for TTP0042 activity is higher than 50 °C, which is the limit for thermostable IVTT (Fig. 2), reactions were first incubated at 50 °C for 2 h for protein synthesis and then, the temperature was increased to 70 °C for 17 h. As shown in Fig. 4, we detected a prominent β–glucosidase activity in the presence of its specific substrate FDGlu and a low-level cellobiose hydrolase activity when the reaction was performed using FDC. We observed background activity when the reactions were performed using the empty plasmid as a template, likely due to autohydrolysis or the presence of other glycosidases in the S30 extracts. These results demonstrate that, given an adequate substrate, enzyme activity assays can be coupled to thermostable IVTT under the tested conditions.

### Encapsulation in water-in-oil droplets

**Fig. 5:**
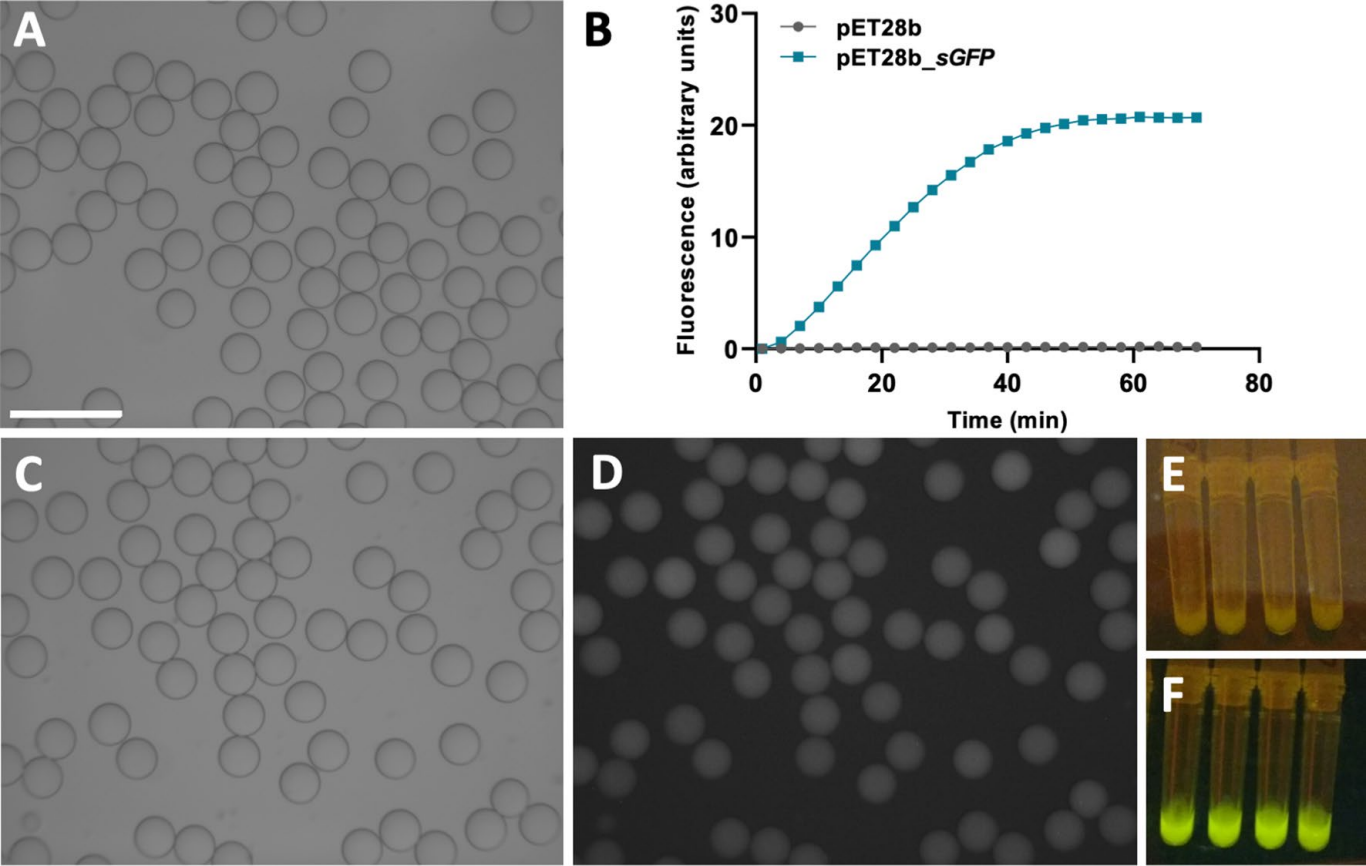
Cell-free protein synthesis in water-in-oil microfluidic droplets. **A**. Reaction mixtures containing 30 ng/µl of pET28b or pET28b_*sGFP* were encapsulated at 50 μl·h-1 using 30 µm flow focusing chips with 500 μl·h-1 1% fluorosurfactant in HFE7500 as the continuous phase. **B**. The droplets containing pET28b_*sGFP* (squares), averaging 34 µm in diameter, were incubated for at 50 ºC for 70 min using a real-time thermocycler. As a negative control of the reaction, droplets containing an empty pET28b vector were used (circles). sGFP synthesized was monitored in real time as fluorescence emission. **C.** Droplets were imaged in an Olympus epifluorescence microscope using brightfield illumination. **D.** or a FITC filter set. **E.** Cell-free protein synthesis with the pET28b template rendered emulsions that were clearly distinguishable from **F.** emulsions with pET28_*sGFP* under blue light. Scale bar: 100 µm

Cell-free extracts could be successfully encapsulated in water-in-oil droplets using PDMS flow focusing chips with standard designs. Several combinations of oils and surfactants were tested for compatibility with the viscosity, salt content and functionality of the IVTT reaction mix. The use of 1–1.5% RAN 008 fluorosurfactant in HFE 7500 provided the best droplet stability during both flow focusing and incubation. As shown in Fig. 5, droplets were monodisperse prior to and after 70 min incubation at 50 °C, successfully achieving thermostable in vitro synthesis of sGFP in water-in-oil droplets for the first time, to the best of our knowledge.

## Discussion

In this study, we describe for the first time a cell-free system for protein synthesis at high temperatures coupled to enzymatic assays and functional in microfluidic droplets. Compared with their mesophilic counterparts, cell-free extracts from thermophilic organisms provide increased protein synthesis rates and substrate solubility, facilitate folding of proteins from thermophiles and reduce the formation of mRNA secondary structures as well as microbial contamination [38]. One of the most popular IVTT solutions (NEB PURExpress^®^) is not fully functional at any temperature above 37 ºC, with a yield reduction of approximately 50% at 40 ºC, compared to 37 ºC and no protein synthesis at 45 ºC or 50 ºC (Supplementary Fig. 3). Therefore, there is a clear technological gap for IVTT at a higher temperature range.

A general advantage of IVTT is the use of a PCR fragment as template, as long as it harbors the necessary regulatory elements. However, we observed a higher efficiency when using plasmid DNA as a template, compared to a PCR product (Fig. 1). This is in agreement with the reported degradation of DNA linear templates for cell-free expression by exonucleases naturally present in cellular extracts, primarily exonuclease V, encoded in the *recBCD* operon [39] and endonuclease I, encoded by *endA* [40]. In order to increase the stability of linear templates, IVTT extracts can be obtained from *T. thermophilus* knockout mutants in addAB [41] or from strains where endonucleases V and I have been silenced before harvesting [42]. Other solutions that do not involve strain customization include adding exonuclease inhibitors to the reaction mix [43], depleting exonuclease V in the crude extracts [44] or protecting the ends of the linear dsDNA template with chemical modifications [45].

Regardless of the type of template, in order to use the *Thermus* IVTT extracts for library screening applications, a single DNA molecule should be amplified in order to yield sufficient protein for accurate detection in the short signal integration times required for ultrahigh-throughput screening [46]. The amplification step will also facilitate the recovery of the coding DNA from a relatively low number of selected droplets after the screening. Therefore, we performed RCA and IVTT in a stepwise manner due to the different temperatures of both reactions. However, we observed an incompatibility between the RCA reaction and *T. thermophilus*-based extracts, although coupling was possible with *E. coli* extracts (Supplementary Fig. 2A) and pure component IVTT system [21]. Whether the incompatibility resides in the highly branched nature of the products [47] or in an unsuitable composition of the reaction medium is unclear. In the future, alternative isothermal amplification methods could be considered, such as helicase-dependent Amplification (HAD) [48], recombinase polymerase amplification (RPA) [49] or the more processive T4 replisome [50].

Regarding the performance of the *Thermus* IVTT extracts used in this work, approximately 60 ng/µl of sGFP were obtained after around 1 h of reaction under optimal conditions. Thus, the methodology proposed in this work renders higher protein concentrations and has better space–time yield than most of the reported solutions for thermostable CFPS (Table 2). In fact, 115 ng/µl can be obtained using *T*. *kodakaraensis* IVT lysates, only after heavy optimization of the lysate preparation process and the strain used [51]. Moreover, this lysate is highly specific for native *T*. *kodakaraensis* genes as evidenced from the poor yield in the synthesis of heterologous GFP, even after codon optimization [52]. Comparing within the same species, our yield is comparable to the reconstituted *Thermus thermophilus* pure component system for IVT, which requires a complex preparation procedure involving the overproduction and purification of 33 recombinant proteins, ribosomes and total tRNAs [12].

**Table 2:** Performance of several CFPS systems under maximum yield conditions

Type of CFPS	Organism and type of extract	RNA polymerase	Temp. (°C)	Template		Synthesized product	Product concentration (ng/μl)	Space–time yield (ng/μl/min)	Refs
				Type	Concentration (μg/ml)				
IVT	*S30 Sulfolobus*	n.a	75	ORF104 RNA	60	Sso ORF 104	n.d	n.a	[15]
IVTT	*S30 Sulfolobus*	None	70	plasmid DNA	250	Sso alkylguanyl transferase	n.d	n.a	[73]
IVTT	S30* Thermococcus*	thermostable T7 RNApol	40, 65	plasmid DNA	67	chitinase from Tkod	0.7	0.005	[65]
IVT	S30 *Thermococcus*	n.a	65	mRNA	400	chitinase from Tkod	115.4	1.91	[51]
IVT	S30* Thermococcus*	n.a	60	mRNA	400	heterologous tGFP	6.5	0.11	[52]
IVT	S30* Thermus*	n.a	65	MS2 phage RNA	100	proteins encoded in MS2 phage RNA	52	0.24	[74]
IVT	S30* Thermus*	None (direct translation)	65	M13mp19 ssDNA	300	poly(Phe)	35	0.16	[35]
IVT	PURE* Thermus*	n.a	65	mRNA	200	stGFP	60	0.50	[12]
IVTT	*S30 Thermus*	thermostable T7 RNApol	50	plasmid DNA	40	sGFP	60	1.08	This work
IVTT	PURE* E. coli*	T7 RNApol	37	plasmid DNA	10	DHFR, λ lysozyme, GFP, GST, T7 gene 10	160	2.67	[11]

*n.a* not applicable, *n.d* not determined

Considering the optimal growth temperature of *T. thermophilus* of approx. 72 °C and the fact that the reconstituted IVT system from *T. thermophilus* is functional up to 65 °C, we attributed the upper temperature limit of the IVTT reaction to the limited stability of the tT7 RNApol compared to the rest of the components. In fact, the tT7 RNApol used in this study shows an optimal temperature of approximately 50 °C with a half-life of 85 min at that temperature and no activity above 50–52 °C, according to the manufacturer. For this reason, we tried to increase the performance of IVTT by increasing the amount of RNA polymerase (Supplementary Fig. 1), albeit unsuccessfully. Therefore, more thermostable RNA polymerases are clearly needed, whether variants of the T7 RNA polymerase or RNA polymerases from thermophilic organisms [53, 54]. An example of the latter would be the RNA polymerase of *Geobacillus sp.* GHH01, which is stable and active up to 55 °C and recognizes DNA template sequences from a wide variety of organisms [55], which is an asset in the activity-based screening of metagenomic libraries. Also, the addition of helper molecules such as *T. thermophilus* chaperones DnaK/ClpB and homologs of GroEL [56, 57], compatible solutes [58–60] or stabilizers, such as trehalose, may increase the stability of the RNA polymerase. Ameliorating the temperature limitation imposed by transcription would also improve the overall yield of the IVTT system, as the translation machinery would be able to function at temperatures closer to the optimal growth temperature of *Thermus.*

Another key issue towards the viability of CFPS is the process of energy generation, which represents the major cost factor and a yield-limiting component. Generally, and also in our extract composition, the supply of ATP is generated from a molecule containing high-energy phosphate bonds, such as phosphoenolpyruvate (PEP), which generates ATP by a substrate-level phosphorylation reaction. However, ATP and PEP have limited stability at high temperatures [61, 62]. They can be degraded by nonspecific phosphatases present in the cell extract [63] and generate the accumulation of inorganic phosphate, which has an inhibitory effect on protein synthesis [64]. These considerations point towards energy depletion as a possible limitation of the yield of the thermostable IVTT, in accordance with the literature [65].

Moreover, high-energy compounds are expensive, representing more than 50% of the total cost of the reaction [66]. This represents an obstacle towards the adoption of IVTT for high-throughput screening, which can be circumvented by operating in microfluidic droplets, reducing the cost of reagents by orders of magnitude [17]. To this end, droplets need to remain stable during the incubation time necessary to carry out the reaction and the environment generated must be biocompatible with the IVTT formulation. In agreement with other works that have used either extract-based [67] or pure component IVTT formulations from *E. coli* [68], we did not observe inactivation or accumulation of proteins at the interface. Moreover, the oil and surfactant mixture is similar to previously reported of IVTT reactions in droplets using the machinery from *E. coli* (HFE7500 perfluorinated oil and 2% v/v PEG-based perfluorinated surfactant), where droplets were subjected to the thermal stress of a PCR amplification step prior to IVTT [69]. In our case, given the milder incubation conditions compared with PCR cycling, 1.5% v/v surfactant proved enough to maintain droplet stability and monodispersity throughout the experiment, as shown in Fig. 5, avoiding undesirable transport effects intrinsic to the increase of surfactant concentration [70].

Finally, we validated the applicability of *T. thermophilus* extract-based IVTT towards functional screenings by coupling IVTT at high temperatures with fluorogenic enzymatic assays. Although we have demonstrated that such reactions are possible with two different examples, the background reaction and autohydrolysis of substrates at higher temperatures will ultimately determine which enzymatic activities can be successfully coupled with thermostable IVTT. If necessary, high levels of background activities can be overcome by deletion of the relevant interfering genes [23], particularly as more genome editing tools become available for thermophilic microorganisms [71].

## Conclusions

Currently, there are no commercial solutions to thermostable CFPS, causing researchers to make their own pure component or cell-based extracts for IVTT. The cell-free extracts from *Thermus thermophilus* described herein represent a simpler alternative to heavily optimized or pure component thermostable in vitro expression systems. The protocol was simple and adaptable to different *Thermus* strains, with yields comparable to the alternative systems mentioned above. Moreover, due to its compatibility with microfluidic droplets and enzymatic assays at high temperatures, the reported IVTT system represents a convenient gateway for enzyme screening at higher temperatures with ultrahigh-throughput. This approach can be used to find thermostable enzyme variants for biocatalysis [72] or novel thermostable enzymes in metagenomic libraries from thermal environments. Finally, this concept can be extended to other types of extreme environments, simply by using IVTT extracts from other extremophiles, opening new avenues for enzyme discovery and evolution as well as synthetic biology of mesophiles and extremophiles.

### Supplementary Information

Supplementary material 1.

## Data Availability

The datasets used and/or analysed during the current study are available from the corresponding author upon reasonable request.
